# Recent Advances of Optical Sensors for Copper Ion Detection

**DOI:** 10.3390/mi13081298

**Published:** 2022-08-11

**Authors:** Zeynep Gerdan, Yeşeren Saylan, Adil Denizli

**Affiliations:** Department of Chemistry, Hacettepe University, 06800 Ankara, Turkey

**Keywords:** copper detection, ion detection, optical sensor, microfluidic, environmental applications, medical applications

## Abstract

A trace element copper (Cu^2+^) ion is the third most plentiful metal ion that necessary for all living organisms and playing a critical role in several processes. Nonetheless, according to cellular needs, deficient or excess Cu^2+^ ion cause various diseases. For all these reasons, optical sensors have been focused rapid Cu^2+^ ion detection in real-time with high selectivity and sensitivity. Optical sensors can measure fluorescence in the refractive index—adsorption from the relationships between light and matter. They have gained great attention in recent years due to the excellent advantages of simple and naked eye recognition, real-time detection, low cost, high specificity against analytes, a quick response, and the need for less complex equipment in analysis. This review aims to show the significance of Cu^2+^ ion detection and electively current trends in optical sensors. The integration of optical sensors with different systems, such as microfluidic systems, is mentioned, and their latest studies in medical and environmental applications also are depicted. Conclusions and future perspectives on these advances is added at the end of the review.

## 1. Introduction

The copper (Cu^2+^) ion is one of the most important metal ion and has a crucial part in many applications with very important functions in nature and the human body [1,2]. It plays an important role in the human body as a catalytic cofactor of redox-regulating enzymes such as for a diversity of metalloenzymes, including tyrosinase, lysyl oxidase, cytochrome c oxidase, and superoxide dismutase [3,4]. It is an indispensable catalyst in the absorption of iron and the synthesis of ferroheme [5]. Cu^2+^ ion does not have a toxic effect on the human body under normal conditions; however, excess and deficiency can cause various harms. Although trace amounts of Cu^2+^ ion are sufficient for the normal physiological process, intake above the required daily dose has negative consequences for human health [6,7].

In addition to the iron metabolism regulated by copper ions, many diseases occur with the increase of concentration due to a lack of this element or long-term exposure. Excess Cu^2+^ ion intake can cause complaints such as diarrhea, vomiting, dizziness, and stomach ache [7]. Free Cu^2+^ ion that has accumulated in the body as a result of long-term exposure produce reactive oxygen species due to redox activity and damage lipids, DNA, and proteins. Free oxygen species are potentially toxic to cells [8,9]. Many diseases may develop in the body depending on the defect in hemostasis. Increasing the concentration of Cu^2+^ ion causes many adverse health problems such as Wilson’s, Parkinson’s, Menkes, neurodegenerative and Alzheimer’s diseases [10,11,12], and also Huntington’s, and acute hepatic kidney failure [13,14,15]. According to the Environmental Protection Agency (EPA), the upper limit for Cu^2+^ ion in drinking water is 1.3 ppm [16,17]. The amount of copper concentration that should be in the blood serum of healthy individuals is 100–150 μg/dL [18,19]. Moreover, Cu^2+^ ion is released into the environment through domestic, industrial, and agricultural processes [20]. Cu^2+^-combined pesticides are commonly utilized to supplement agricultural growth and prohibit diseases. Since 1991, the EPA has restricted the level of Cu^2+^ ion in tap water to 20 μM [21], and the World Health Organization (WHO) has regulated drinking water at 30 μM [22]. Freshwater, agricultural products, soil, drinking water, and sediment can be contaminated because of the excess use of these pesticides.

**Figure 1:**
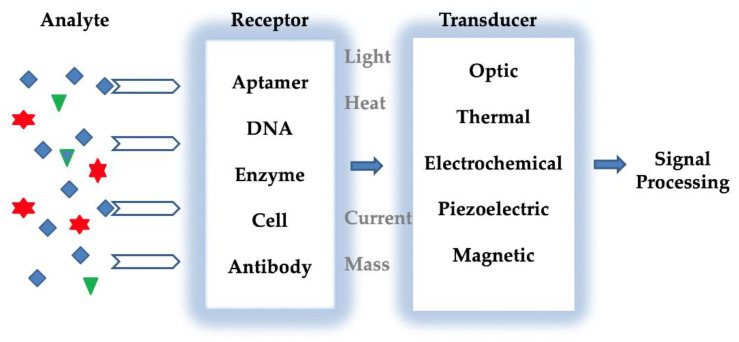
Typical detection principle of a biosensor.

Various methods are used to detect trace metals. In Cu^2+^ ion detection, atomic absorption spectrometry [23,24,25], anodic stripping voltammetry [26,27], high-performance liquid chromatography, inductively coupled plasma atomic emission spectrometry [28,29,30], and inductively coupled plasma mass spectrometry [31] are widely used. However, these methods have advantages, such as being reliable, accurate, and fast, as well as disadvantages, such as being expensive and complex processes, which must be overcome [32,33,34]. It is essential and immediately to improve sensitive, easy, rapid, reproducible, low-cost, and analytical methods for Cu^2+^ ion detection in environmental and medical samples [35]. In this regard, sensors are analytical devices that convert physical, chemical, and biological changes in the environment into electrical signals [36,37]. Generally, these sensors consist of three main parts: transducers, receptors, and electronic parts [38,39]. A typical biosensor is shown in Figure 1. The sensing principle is based on the specific interaction between the analyte and receptor. Depending on the interaction, properties such as changing pH, electron, mass transfer, temperature, a change of optical properties, and potential differences are detected by the transducers. This system converts the receptor’s response into an electronic signal that is directly related to the existence of the analyte or commensurate to the analyte concentration [40]. The increase and interest in sensor studies in recent years is due to their low cost, easy miniaturization, and production of semi-quantitative information in a short time. For all these reasons, sensors have become very significant tools for clinical environmental and food monitoring and detection of chemical and biological compounds [41,42,43]. The analyte used in sensor applications is defined as the substances and structures to be analyzed. Receptors are elements that make-up compounds or mixtures. The most common receptors are enzymes and antibodies, but in general, polymers, dyes or chelating agents are also used for sensor surface modification [44]. Transducers are part of sensors that convert the sensed energy from one form to another [45,46]. The selectivity of the sensors is a very important parameter, as the sensors must respond to analytes in complex matrices of real samples [47]. In sensors, transducers such as electrochemical, optical, and piezoelectric are used during the measurement and transmission of the signal formed as a result of the interaction between samples and ligands [48]. Thus, sensor technology is a widely used range of platforms such as biomedical, health technologies, pharmacological and environmental analysis [49,50,51].

## 2. Optical Sensors

Optical sensors are highly sensitive and selective to the analyte to be analyzed. They have gained great attention in recent years due to the excellent advantages of simple and naked eye recognition, real-time detection, low cost, high specificity against analytes, their quick response, and less complex equipment in analyses [52,53,54]. Optical sensors have countlessly subdivided according to the change in the signal resulting from the interaction between the recognition element and target molecule. The subdivisions are refractive index, fluorescence, chemiluminescence, infrared spectrum, colorimetric, and Raman spectra [55,56,57,58,59,60]. Nowadays, optical sensors have proceeded to develop in many applications including food safety [61], virus detection [62], cancer diagnosis [63,64], cardiac biomarkers detection [65], environmental monitoring [66], DNA sensing [67], and blood glucose monitoring [68]. 

There are several kinds of optical sensors in the literature with different platforms. For instance, the technology of processes with small amounts of fluids (up to 10^−18^ L) utilizing channels called microfluidics [69,70]. The microfluidic platforms have the potential to alter subject areas from analysis to information technology. The first applications of microfluidics aim to have many helpful superiorities including the capability to employ low amounts of samples and to carry out detections with high resolution, short time, and low cost [71,72]. Microfluidics works through its most obvious properties such as its small size and less visible properties of fluids in microchannels. It proposes fundamentally novel abilities in the control of concentrations of biomolecules [73].

### 2.1. Colorimetric Sensors

Colorimetric sensors are used to detect the instantaneous modification in color that occurs as a result of the interaction between the target analyte and reacting sensing element [74,75]; this color change is seen by the naked eyes. The main purpose of colorimetric sensors is how stimuli such as pH values, temperature change, and stress cause a visible change in the analyte [76,77,78]. Colorimetric sensors have many advantages, such as naked eye detection, low capital cost, utmost simplicity, good selectivity and specificity, short-time analysis, reversibility, and lacking the need for the requirement of complex instruments [79,80,81]. There are several colorimetric sensors for Cu^2+^ ion detection. 

For instance, Gangapuram et al. improved gold nanoparticles (AuNPs)-modified colorimetric sensor for the selective and sensitive Cu^2+^ ion detection. Synthesized AuNPs were characterized and they showed excellent stability in different conditions. Carboxymethyl gum karaya capped with AuNPs demonstrated a selective colorimetric response with a visible change from red to blue. These results were approved by TEM and DLS analysis. This assay showed a well linear correlation in the range of 10–1000 nM of Cu^2+^. The limit of detection was calculated as 10 nM. Tap water, human plasma, and human urine samples were used to evaluate the detection capability of this sensor [82]. 

**Figure 2:**
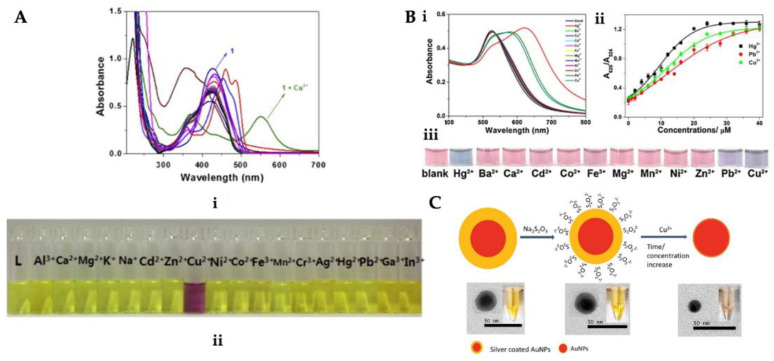
(**i**) Absorption and (**ii**) color changes upon the addition of different metal ions (**A**), (**i**) UV-vis absorption spectra with distinct metal ions, (**ii**) P-AuNPs against several concentrations of Cu^2+^, Hg^2+^, and Pb^2+^ ions (**iii**) solution with different metal ions (**B**) and scheme of the Ag/AuNPs for Cu^2+^ ion detection (**C**). Republished with permission from [83,86,87].

For the detection of Cu^2+^ ion, Park et al. also prepared a receptor 1. The chromogenic perception capability of receptor 1 was examined in the asset of 18 different cations. Figure 2(Ai) shows the absorption spectra changed only when Cu^2+^ induced, and Figure 2(Aii) depicts the solution color of receptor 1 changing from yellow to purple with the addition of Cu^2+^ ion [83]. Deng and colleagues developed a colorimetric sensor to detect Cu^2+^ with gold nanoparticles. The existence of Cu^2+^ ion causes the color to alter from red to purple–blue. The limit of detection was determined as 0.04 μM with the UV-Vis spectrometry and 2 μM with the naked eye [84]. A colorimetric hydrazone-based ligand was developed for Cu^2+^ ion detection by Abdulazeez et al. This developed ligand did not show the selectivity for Cu^2+^ ion against different metals and a color change was only observed in the prepared Cu^2+^ ion solution. In other metals, the color change was insignificant or not at all. Ligand showed good selectivity and sensitivity. The detection limit value was reported as 0.34 μg/L [85]. 

Guo et al. presented an easy colorimetric sensor to determine Cu^2+^, Hg^2+^, and Pb^2+^ ions (Figure 2B). Cu^2+^, Hg^2+^, and Pb^2+^ ions were dropped into papain-coated gold nanoparticles (P-AuNPs) solution prepared for colorimetric detection. The P-AuNPs showed different responses to Cu^2+^, Hg^2+^, and Pb^2+^ ions in an aqueous solution. They examined the concentration, pH, and size impact on the sensitivity and stability of the sensor. To analyze the selectivity of the sensor, the colorimetric reaction was explored in the existence of diverse metals. The color intensity of the adsorbent enhanced with an increase in the Cu^2+^ concentration leading to the red-shift of the peak at 375 nm and also the emergence of a novel peak at about 490 nm by UV-vis-NIR spectroscopy [86]. Lou et al. synthesized silver-coated gold nanoparticles (Ag/AuNPs) for Cu^2+^ ion detection in water samples (Figure 2C). The leaching of Ag/AuNPs would cause a fair decrease in the surface plasmon resonance absorption as the dimensions of Ag/AuNPs decreased. This colorimetric sensor is dependent on the dimensions-based nanoparticles’ highly sensitive and selective sensing toward Cu^2+^ ion. They reported that this sensor ensured an easy and fast platform for heavy metal detection [87].

Xei et al. prepared a microfluidic system-integrated colorimetric sensor for Cu^2+^ ion detection. They observed that in the presence of Cu^2+^ ion, the channel color values decreased with increasing the Cu^2+^ concentration, and also, the intensity was linear in the range of Cu^2+^ ion concentration (0–30 mg/L) with a limit of the detection value of 0.096 mg/L. They also performed tap water analysis with this microfluidic system-integrated colorimetric sensor and observed high selectivity and recoveries and also, satisfying reproducibility. Additionally, by changing a hole punch with different shapes and numbers, it is very easy to produce sensors with different designs at a low cost [88]. 

### 2.2. Fluorescence Sensors

Fluorescence sensors are widely used for sensing different molecules. They do not need high excitation power [89]. The fluorescence has quite high sensitivity and selectivity. The fluorescence sensors observe the change of frequency of electromagnetic radiation emission induced by preceding radiation absorption and excited state generation that only rises for a very limited time [90]. Fluorescence sensors have advantages such as less response time with high selectivity and sensitivity and have applications in environmental monitoring, microscope-based analysis, in clinical diagnostics and food safety; they are broadly employed for the detection of heavy metal ions [91,92]. These advantages have proven to be the most appropriate approximation for metal ions detection [93]. Fluorescence detection of Cu^2+^ ion is more difficult than many other metals. The reason for this difficulty is due to the paramagnetic nature of Cu^2+^ ion, which has inherent quenching properties of Cu^2+^ ion [94,95]. 

**Figure 3:**
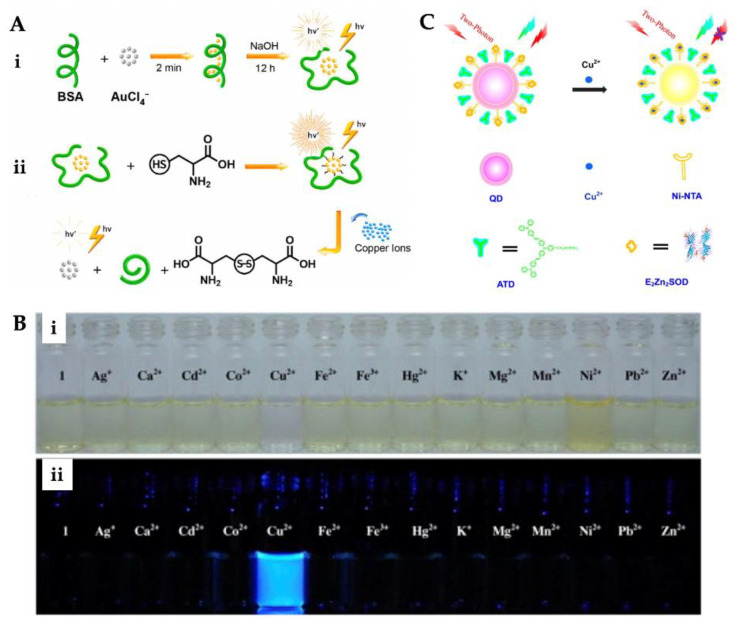
Preparation steps of fluorescence sensor for Cu^2+^ ion detection (**A**), color (**i**) and fluorescence (**ii**) changes of sensor after addition of various metal ions (**B**). Scheme of the working principle of two-photon ratiometric imaging and sensing of Cu^2+^ ion (**C**). Republished with permission from [98,99,100].

For example, Liu et al. fabricated a fluorescence sensor for extremely sensitive Cu^2+^ ion detection. The surface of silica-coated CdSe quantum dots was conjugated to the Cu^2+^ ion nanoclusters and they designed the fluorescence sensor. In the asset of various quantities of Cu^2+^ ion, a color shift from yellow–green to red was observed in the sensor. The detection limit for Cu^2+^ ion was calculated to be 8.9 nM [96]. Xie and co-workers reported the quantum dots acted as a fluorescence sensor to distinguish and determine Cu^2+^ and Ag^2+^ ions. They observed bright yellow fluorescence emission under a 365 nm UV lamp and absorption at 525 nm. With this method, detection of Cu^2+^ and Ag^2+^ ions takes only a few minutes. The method can carry out the differentiation of Cu^2+^ and Ag^2+^ ions by principal components analysis plots; the detection limit was 35 nM, and this sensor also has good trustworthiness and correctness in real samples [97]. A switchable fluorescence sensor was improved for the determination of Cu^2+^ ion by Niu et al. (Figure 3A). This method showed good linearity for Cu^2+^ ion under optimum conditions. The fluorescence sensor in the search of Cu^2+^ ion showed a peak (emission) at 660 nm upon peak (excitation) at 495 nm. As a result of the experiments on living samples, the fluorescent sensor’s rapid, high stability, and sensitivity were promising for diagnosing Alzheimer’s [98]. Wang et al. prepared a pyrene-based fluorescence sensor for Cu^2+^ ion determination. In the asset of Cu^2+^ ion, the sensor ensured an important fluorescence increase. Maximum fluorescence increase was observed with the binding of Cu^2+^ ion to the sensor at the range of pH 2.0–8.5. Selectivity analyzes of the prepared fluorescence sensor were performed using Ag^+^, Ca^2+^, Cd^2+^, Co^2+^, Fe^2+^, Fe^3+^, Hg^2+^, K^+^, Mg^2+^, Mn^2+^, Ni^2+^, Pb^2+^, and Zn^2+^ metal ions. As depicted in Figure 3B, Cu^2+^ ion caused a visible color change in the sensor from light yellow or colorless and had a blue emission [99].

Fu et al. designed a two-photon fluorescence sensor for imaging and sensing Cu^2+^ ion (Figure 3C). The selectivity of the fluorescence sensor was examined employing Na^+^, K^+^, Ca^2+^, Mg^2+^, Mn^2+^, Fe^2+^, Co^2+^, Ni^2+^, Zn^2+^, and Cu^+^ metal ions. The detection was achieved down to around 10 nM in a wide range (10^−3^–10^−7^ M) [100]. Peng et al. developed a fluorescence sensor containing two DNA sequences. The prepared sensor showed high selectivity for Cu^2+^ ion over various other metals. River water samples were analyzed by the standard addition method. The detection limit of the hybridized double-strand fluorescence sensor was calculated as 3.4 nM [101]. Furthermore, Du et al. designed a metal–organic framework-based fluorescence sensor for Cu^2+^ ion detection in aqueous conditions. The assess the ability of the sensor to determine Cu^2+^ ion, fluorescence quenching studies were made with distinct Cu^2+^ ion concentrations. The ability of selectivity and sensitivity experiments were also conducted. The results displayed that the designed fluorescence sensor could be a promising platform for real-time monitoring and bifunctional intelligent adsorbent Cu^2+^ ion detection with 1.91 × 10^−7^ M of detection limit [102]. Xie et al. prepared a fluorescence sensor for the determination of Cu^2+^ ions in tea infusions. The fluorescence sensor was prepared for homogeneous precipitation of CdS nanocrystals onto the SiO_2_ core surfaces. According to the selectivity analysis, the fluorescence sensor was more sensitive to Cu^2+^ ion than other metal ions. The reason for this is the strong fluorescence quenching effect of Cu^2+^ ion. The sensor determined the Cu^2+^ ion in extensive linear ranges from 0.01 to 2 μM and the detection limit was calculated as 6.3 nM [103]. Tan et al. reported a fluorescence sensor for Cu^2+^ ion detection in serum samples. Carbon dots and gold nanoclusters were embedded into ZIF-8 and the potential guideline of the sensor for detecting Cu^2+^ ion was exemplified. The synthesized sensor’s characterization by TEM and detection performance was optimized [104]. 

Chatterjee et al. designed an anthracene excimer fluorescence sensor on mesoporous silica, which has important advantages such as chemical stability and a large surface area. The structural characterization of cubic mesoporous silica was performed with several methods, and the reusability of materials was analyzed. This sensor material was analyzed in orange and grape juice samples for Cu^2+^ ion detection. In line with the tremendous results obtained, the sensor presents a very uncommon sample of an excimer-based heterogeneous sensor [105]. Cheng et al. also presented a fluorescence sensor in which Cu^2+^ ion was detected in an aqueous solution and in living cells. For rapid detection of Cu^2+^ ion in living organisms, metal–organic framework nanoparticles have unique physical and chemical properties with an easy and environmentally friendly hydrothermal route. The high affinity between Cu^2+^ ion and the porphyrin ligand in the structure of metal-organic framework nanoparticles can statically quench the fluorescence signal of the sensor with Cu^2+^ ion with high selectivity. The prepared sensor has an ultra-low limit of detection value of 220 pM [106]. Patir et al. reported nitrogen-doped carbon dots fluorescence sensor that was prepared by a one-step pyrolytic method utilizing urea and ethylenediaminetetraacetic acid for Cu^2+^ ion detection. The lowest detection limit for Cu^2+^ ion detection was 2.3 nM in an aqueous medium, which is close to the allowed levels of Cu^2+^ ion in drinking water. They loaded a paper-based microfluidic system loaded with nitrogen-doped carbon dots using candle wax channels on a paper. They mentioned that this sensor system is low-cost, simple and disposable paper-based platform will be very helpful for onsite detection [107].

### 2.3. Luminescence, Chemiluminescence, and Photoluminescence Sensors

**Figure 4:**
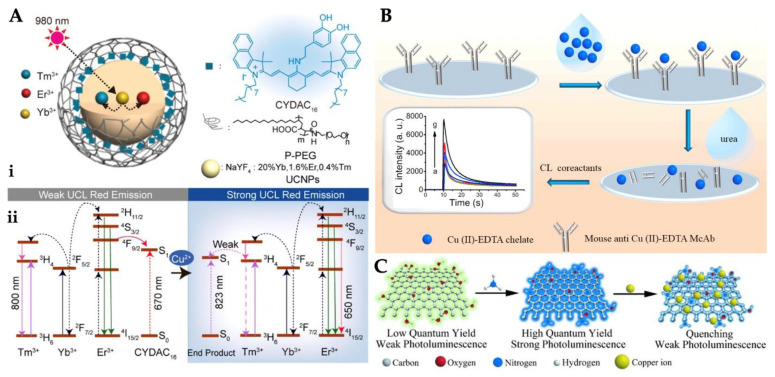
Unification of nanoparticles and CYDAC_16_ (**A**), scheme of the protocol for Cu^2+^ ion detection (**B**) and representation of amino-functionalized quantum dots and their quenching by Cu^2+^ ion (**C**). Republished with permission from [109,115,116].

Luminescence occurs when an excited molecule emits light as it returns to its lower energy level ground state. A few types of luminescence can be sundered base on the welding of the energy that cause an excited state. Chemiluminescence is an emission of light that is dependent on chemical reactions and is of the greatest interest to researchers [108]. As an example, Shi et al. depicted a luminescence sensor for Cu^2+^ ion determination. The nanoparticles selected for radiometric imaging were combined with a fluorescence sensor CYDAC_16_. The interaction between nanoparticles CYDAC_16_ and Cu^2+^ ion was explained in Figure 4A. The nanoparticle CYDAC_16_ ensures a ratiometric signal based on an up-conversion luminescence of 660 and 800 nm. The sensor performed an analysis of living mice and cells and could accomplish Cu^2+^ ion detection with the luminescence resonance energy transfer. The detection limit was calculated as 37 nmol/L [109]. Li et al. constructed a luminescence sensor offering high sensitivity and selectivity in the determination of Cu^2+^ ion. COF-JLU3 synthesized under solvothermal conditions has a porous framework structure that mediates the binding of Cu^2+^ ion. In the existence of Cu(NO_3_)_2,_ the fluorescence lifetime decreased from 1.5 ns to 0.7 ns. The reason for this is the decrease in luminescence intensity [110]. 

Chemiluminescence transition is a chemical reaction excited with the emission of light in the upper state while returning to the ground state. These transitions are defined as electromagnetic radiation dissipated from the near-ultraviolet to the near-infrared [111,112]. Chemiluminescence, which is the production of light from a chemical reaction, has many advantages, such as simplicity, rapidity, and sensitivity of detection [113,114]. For instance, Ouyang et al. reported a chemiluminescence sensor for facile, fast, sensitive, and affordable cost for the determination of Cu^2+^. As depicted in Figure 4B, Cu(II)-EDTA was used as a chelate and then immobilized onto the microplate. They computed the detection limit value as 0.33 ng/mL, and this value obtained a wide range of 1.0–1000 ng/mL [115].

To show the precise detection of Cu^2+^ ion and a stronger enhancing effect, gold nanostars were utilized for chemiluminescence sensor preparation by Amjadi and Abolghasemi-Fakhri. To increase density, nanostars were produced using seed-mediated growth. Attaching the tips of nanostars to surface plasmons causes an increase in emission intensity. The gold nanostar-based sensor system can detect very low levels in the sensitive and fast detection of Cu^2+^ ion [117]. Moreover, Chan et al. presented a photoluminescence sensor for highly sensitive Cu^2+^ ion detection. 16-Mercaptohexadecanoic acid-modified CdSe quantum dots were prepared as a sensor. Research on the presence of very low concentrations of Cu^2+^ ion showed a reduction in the sensor of quantum dots. They calculated the limit of detection as 5 nM in the dynamic range of up to 100 µM, and also the reaction time was observed at 5 min [118]. Wang et al.’s photoluminescence sensor was developed for determining Cu^2+^ ion. A cellulose nanofibril was produced by the in situ synthesis procedure. Fluorescence spectrometer, Fourier transforms infrared spectroscopy, scanning electron microscopy, and X-ray diffraction were used for the characterization of nanofibril film samples. Depending on the increment in the Cu^2+^ ion concentration, there was a gradual decrease in fluorescence intensity of cellulose nanofibril, and a linear relationship was determined between them and the Cu^2+^ ion [119]. 

Wang et al. prepared carbon dots with different solutions for Cu^2+^ ion detection by the hydrothermal method. The concentration-dependent multicolor photoluminescence emitted different colors with decreasing concentration, exhibiting the three strongest peaks. This sensor was highly selective and sensitive to detect Cu^2+^ ion at ppm limits [120]. Sun et al. designed a fluorescence sensor for the determination of Cu^2+^ ion in living cells depending on the hydrothermal treatment of graphene quantum dots. Following this process, the greenish-yellow fluorescent graphene quantum dots were transformed into amino-functionalized graphene quantum dots (Figure 4C). Compared with other metal ions, Cu^2+^ ion have a greater affinity for N and O on the amino-functionalized graphene quantum dots’ surface, thus, the chelating kinetics is faster. Amino-functionalized graphene quantum dots also showed higher selectivity towards Cu^2+^ ion [116]. Ganiga et al. designed nitrogen-rich carbon dots to detect Cu^2+^ ions. Their study was the first to employ the interaction of Cu^2+^ ion with NCD-based fluorescent nanomaterials to unravel the photoluminescence conduct. As a result of the exhaustive analyzes made, the steady-state photoluminescence emission of NCDs was revealed from the direct recombination of excitons and the involvement of defect states. The detection limit was calculated as 10 μM in 10 μM–0.4 mM dynamic range, respectively [121].

Zhao et al. prepared a selective fluorescence sensor determination for Cu^2+^ ion. The photoluminescence intensity of the sensor prepared using polydopamine, which does not have any photoluminescence properties, increases even more in the existence of Cu^2+^ ion. The sensor detection limit was determined to be as low as 1 nM [122]. A sensor was designed for Cu^2+^ ion detection by coating carbon dots synthesized by Liu et al. hydrothermal route with branched polyethylenimine. Photoluminescence emission spectra of the carbon quantum dots with an increment of excitation peaks from 365 to 525 nm, emission peaks are red-shifted from 440 to 540 nm. Photoluminescence intensities were diminished. The fluorescence sensor with high selectivity detected Cu^2+^ ion to a low detection limit of 115 nM [123]. Liu et al. developed pristine graphene quantum dots that were produced with oxidation of pitch graphite fibers to detect Cu^2+^ ion. The results showed the photoluminescence properties of these quantum dots could be removed by different metals during additional cysteine that can only cause recovery of the photoluminescence of graphene quantum dots removed by Cu^2+^ ion [124].

### 2.4. Surface Plasmon Resonance

Surface plasmon resonance (SPR) is an important optical sensor type that depends on the power of reflected light from a prism that is covered with a metal film [125,126]. SPR sensors use surface plasmon (SP) waves to research molecular interactions occurring on the sensor surface [127]. The SP is an electromagnetic wave induced by p-polarized light; this wave is spread along the surface of nanoparticles or layers [128,129]. The area vector of this wave achieves its highest value at the interface and it, therefore, decomposes in both the dielectric and the metal, and it decays exponentially in both environments. This wave generates SP polaritons propagating along the interface [130,131]. SP is not formed directly by light excitation of a flat metal surface. The Kretschmann configuration is commonly used to stimulate plasmons. In this configuration, the prism is in contact with a thin plasmonic metal surface to measure dielectric permittivity [132,133,134]. 

**Figure 5:**
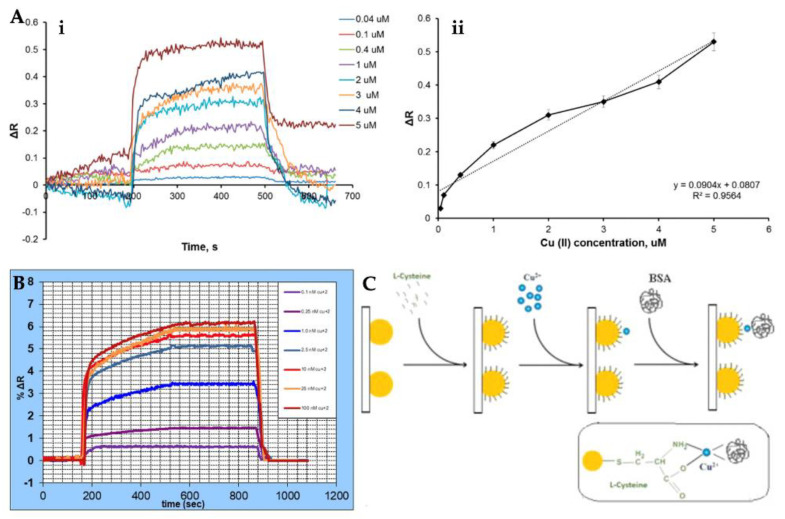
Real-time detection by Cu^2+^-imprinted SPR sensor (**A**), %ΔR values of SPR sensor versus time at several concentrations of Cu^2+^ ion (**B**), and the illustration for LSPR-based Cu^2+^ ion detection (**C**). Republished with permission from [147,148,152].

SPR sensors have many advantages, such as low cost [135], no labeling [136], require a low sample volume, real-time measurement [137], high sensitivity and specificity, and allow fast measurement [138,139,140,141]. They have increasing applications in the detection of various analytes in medical diagnosis, environmental monitoring, food safety, and so on [142,143,144,145,146]. Recently, Gerdan et al. prepared a molecularly imprinted nanofilm-based SPR sensor to detect Cu^2+^ ion in buffer and also artificial plasma and urine samples. The SPR sensor was comprehensively characterized and then used for Cu^2+^ ion detection from solutions with a wide range (0.04–5 μM) in a high correlation coefficient (Figure 5A). They calculated the SPR sensor detected Cu^2+^ ion with a low limit of detection of 0.027 µM. Other kinetic experiments, such as reusability, selectivity, and storage stability, were also performed to show all properties of the sensor [147]. Safran et al. determined Cu^2+^ ion using an SPR sensor. Firstly, they modified a gold sensor surface with poly(hydroxyethyl methacrylate-N-metacryloyl-(L)-cysteine methyl ester and the modified surface was used for the immobilization of Cu^2+^ ion. Characterization measurements of the sensor surface were carried out with different methods. The SPR sensor was also used for the demonstration of the selectivity and sensitivity in aqueous solutions [148]. Figure 5B depicts the %ΔR values of the SPR sensor at different concentrations. 

Forzani et al. also reported SPR sensor to detect Cu^2+^ ion. The surface was separated into reference and detection areas. From these areas, the various angles were measured with a quadrant cell photodetector. The response was changed in the existence of the Cu^2+^ ion. Selective detection of Cu^2+^ ion in the broad range was achieved. Drinking water was analyzed with this sensor [149]. Daniyal and co-workers used the SPR spectroscopy for sensing Cu^2+^ ion. This sensor was made by adding graphene oxide and modifying it on the surface. The detection of the Cu^2+^ ion to the sensor surface was observed by SPR and values were calculated, such as the detection limit and signal-to-noise ratio. The optical sensor detection range was 0.01 until 0.5 ppm [150]. Chen et al. presented a sensor for the detection of Cu^2+^ ion. This sensor is dependent on the conformational change of Cu^2+^-specific peptides. Peptides that bind specifically to the Cu^2+^ ion were modified on the surface. Then, selectivity analyzes of the sensor were performed and the peptide showed good selectivity towards Cu^2+^ ion. The detection limit was calculated as 0.44 pM, and the detection range was 1 × 10^−12^ M to 1 × 10^−6^ M [151]. Finally, Ding et al. designed a sensor that determined Cu^2+^ ion in a real sample. With the self-assembled method, indium tin oxide film-coated gold nanoparticles were prepared (Figure 5C) and then the characterization process was carried out. The strong chelation between Cu^2+^ and Cys, which allows the formation of a stable Cys-Cu^2+^ complex, was formed by modifying the Cys onto the gold surface. Thereby it causes a shift in the LSPR absorption band. The alteration occurring with the red-shift at the peak of the LSPR band is the basis for Cu^2+^ ion detection [152].

## 3. Conclusions

Optical sensors are generally utilized in biomedical and pharmaceutical research, environmental applications, and health care to determine different biomolecules for disease diagnosis. In this review, we overviewed recent and different optical sensing technologies as state-of-the-art Cu^2+^ ion detection applications. Early diagnosis of diseases that develop due to deficiency or excess of Cu^2+^ ion at very low concentrations is very important. The optical sensors are thus attractive because of the advantages of simple and naked eye recognition require less equipment in analysis, real-time detection, high reversibility, environmental stability, durability, and practicability. Cu^2+^ ion detection systems still have some challenges in terms of point-of-care diagnostic procedures for special laboratories and are open to improving optical performance and chemical and physical properties toward more extensive applications in various fields. Moreover, microfluidic systems-integrated optical sensors are proposed as complementary platforms, which refers to conventional methods for the detection of several molecules with rapid response and convenience of usage. In summary, the detection of Cu^2+^ ion is very crucial because it is an important metal for the human body. As depicted in Table 1, there has been an increase in studies on several optical sensors with many parameters, including polymer types, detection range, and limit of detection (LOD) values. The results show that they can be combined with other methods, technologies, and platforms. Among these detection systems, some of them aim at developing the quality and enabling reliability to detect diseases in their early stages and measure food quality for human health.

## Figures and Tables

**Table 1:** Comparison of different optical sensors for Cu^2+^ ion detection.

Ref.	Sensor	Polymer Type	Range	LOD	Selectivity	Real Sample
[82]	Colorimetric	Carboxymethyl gum karaya-capped gold nanoparticles	10–1000 nM	10 nM	Cr^3+^, Zn^2+^, Fe^2+^, Co^2+^, Cd^2+^, Mn^2+^, Ni^2+^, Hg^2+^, Ca^2+^, Mg^2+^, Ag^+^, K^+^	Tap water, human plasma, and urine
[83]	Colorimetric	Julolidine-containing naphthol-based probe	3.0 × 10^−5^ M	1.4 × 10^−5^ M	F^−^, Cl^−^, Br^−^, I^−^, OAc^−^, CN^−^, SCN^−^, SO_4_^2−^, H_2_PO_4_^−^	Not available (NA)
[84]	Colorimetric	Thermally treated gold nanoparticles	0–6 μM	0.04 μM	Zn^2+^, K^+^, Ca^2+^, Na^+^, Mg^2+^, Al^3+^, Fe^2+^, Fe^3+^, Cr^3+^, Cd^2+^, Hg^2+^, Pb^2+^, Ba^2+^, Ag^+^	Mineral water
[85]	Colorimetric	Hydrazone	2 × 10^−3^ M	0.34 μg/L	Mg^2+^, Ca^2+^, Ni^2+^, Zn^2+^,Pd^2+^	NA
[86]	Colorimetric	Papain-coated gold nanoparticles	20 μM	200 nM	Pb^2+^, Ba^2+^, Ca^2+^, Cd^2+^, Co^2+^, Fe^3+^, Mg^2+^, Mn^2^+, Ni^2+^, Zn^2+^	Lake and tap water
[87]	Colorimetric	Silver-coated gold nanoparticles	5–800 nM	1 nM	K^+^, Li^+^, Na^+^, Mg^2+^, Ag^+^, Ca^2+^, Mn^2+^, Al^3+^, Hg^2+^, Cd^2+^, Zn^2+^, Cr^3+^, Co^2+^, Pb^2+^	Tap and pond water
[88]	Colorimetric	Patterned-PVC film	0–30 mg/L	0.096 mg/L	K^+^, Zn^2+^, Ca^2+^, Pb^2+^, Ni^2+^, Mg^2+^, Na^+^, Fe^2+^, Fe^3+^, Co^2+^	Tap water
[96]	Fluorescence	Silica-coated quantum dots	22 nM–8.8 mM	8.9 nM	Cr^3+^, Fe^2+^, Ni^2+^, Co^2+^, K^+^,Ti^2+^, Mn^2+^, Mg^2+^, Ca^2+^, Sn^2+^, Al^3+^, Cd^2+^, Pb^2+^, Hg^2+^, Fe^3+^, Ag^+^	River water
[97]	Fluorescence	Mercaptoacetic acid-coated quantum dots	40–600 nM	35 nM	Ni^2+^, Co^2+^, K^+^, Mn^2+^, Ca^2+^, Na^+^, Al^3+^, Ba^2+^, Cd^2+^, Pb^2+^, Hg^2+^, Fe^3+^, Ag^+^, Mg^2+^	Human urine
[98]	Fluorescence	Bovine serum albumin-stabilized gold nanoclusters	0.5–30 μM	0.1465 μM	Ca^2+^, Mg^2+^, Na^+^, K^+^, Zn^2+^, Sn^2+^, Cr^3+^, Fe^3+^, Fe^2+^, Pb^2+^	Mice
[99]	Fluorescence	Pyrene and hydrazone	50 μM	2.73 μM	Ag^+^, Ca^2+^, Cd^2+^, Co^2+^, Fe^2+^, Fe^3+^, Hg^2+^, K^+^, Mg^2+^, Mn^2+^, Ni^2+^, Pb^2+^, Zn^2+^	Live cells
[100]	Fluorescence	Amino triphenylamine dendron-hybridised quantum dots	10^−3^–10^−7^ M	10 nM	Na^+^, K^+^, Ca^2+^, Mg^2+^, Mn^2+^, Fe^2+^, Co^2+^, Ni^2+^, Zn^2+^, Cu^+^	Live cells
[101]	Fluorescence	Oligonucleotides-stabilized silver nanoclusters	6–240 nM	3.4 nM	Ag^+^, Ca^2+^, Cd^2+^, Co^2+^, Fe^2+^, Fe^3+^, Hg^2+^, Mg^2+^, Mn^2+^, Pb^2+^, Zn^2+^	River water
[102]	Fluorescence	Metal-organic frameworks	2.07 × 10^−7^–8.29 × 10^−4^ M	1.91 × 10^−7^ M	Ca^2+^, Cd^2+^, Co^2+^, K^+^, Mg^2+^, Ni^2+^	NA
[103]	Fluorescence	Silica-anchored nanocrystals	0.01–2 μM	6.3 nM	Al^3+^, Fe^3+^, Ca^2+^, Pb^2+^, Hg^2+^, Mg^2+^, Zn^2+^, Fe^2+^, Cr^2+^, Ba^2+^, Cd^2+^	Tea
[104]	Fluorescence	Carbon dots/gold nanoclusters-embedded metal-organic frameworks	10^−3–^10^3^ μM	0.3324 nM	Na^+^, Fe^3+^, Zn^2+^, Mg^2+^, Fe^2+^, Pb^2+^, Al^3+^, Ca^2+^	Human serum
[105]	Fluorescence	Silica-based hybrid material	1–5 µM	5.44 ppb	Li^+^, Na^+^, K^+^, Ca^2+^, Mg^2^, Sr^2+^, Mn^2+^, Fe^3+^, Co^2+^, Ni^2+^, Zn^2+^, Hg^2+^, Cd^2+^	Grape and orange juice
[106]	Fluorescence	Porphyrinic metal-organic frameworks	1–250 nM	220 pM	Mg^2+^, Zn^2+^, Ca^2+^, Cd^2+^, Fe^2+^, Fe^3+^, Ni^2+^, Ag^+^, Al^3+^, Hg^2+^, Co^2+^, Pb^2+^	Live cells
[107]	Fluorescence	Nitrogen-doped carbon dots	0–25 μM	2.3 nM	Ag^+^, Pb^2+^, Fe^3+^, Cr^6+^, Zn^2+^, Au^3+^, Co^2+^, Hg^2+^	Tap water
[109]	Luminescence	Lanthanide-doped upconversion nanoparticles	12 μmol/L	37 nmol/L	Tm^3+^, Yb^3+^, K^+^, Na^+^, Er^3+^, Cd^2+^, Ca^2+^, Co^2+^, Ni^2+^, Al^3+^, Mg^2+^, Mn^2+^, Zn^2+^, Sn^2+^, Ba^2+^, Hg^2+^, Ag^+^, Fe^3+^, Fe^3+^, H_2_O_2_	Live mice and cell
[110]	Luminescence	Azine-linked covalent organic frameworks	0–0.4 μM	0.31 μM	Li^+^, Na^+^, K^+^, Mg^2+^, Ca^2+^, Ba^2+^, Zn^2+^, Cd^2+^, Ni^2+^, Pb^2+^, Co^2+^, Ag^+^, Fe^3+^, Al^3+^	NA
[115]	Chemiluminescence	Monoclonal antibody	1.0–1000 ng/mL	0.33 ng/mL	Fe^3+^, Pb^2+^, Hg^2+^, Cd^2+^, Ca^2+^, Zn^2+^, Mn^2+^, Cr^3+,^ Co^2+^, K^+^, Na^+^, Ba^2+^, Mg^2+^, Ag^+^, Fe^2+^, Al^+3^, NH_4_^+^	Lake water
[117]	Chemiluminescence	Gold nanostars	0.002–9 μM	0.9 nM	Mn^2+^, Fe^3+^ Pb^2+^, Zn^2+^, Co^2+^, Cr^3+^, Al^+3^, As^5+^, Hg^2+^, Cd^2+^, Eu^+3^, Fe^2+^, Na^+^, Ag^+^, Ni^2+^, Cr^6+^	Human plasma, well and river water
[118]	Photoluminescence	Mercaptohexadecanoic acid-capped quantum dots	0–100 µM	5 nM	Ni^2+^, Mn^2+^, K^+^, Ca^2+^, Co^2+^, Pb^2+^, Na^+^, Ba^2+^	Physiological fluids
[119]	Photoluminescence	Metal-organic frameworks-oxidized cellulose nanofibrils	0–100 µM	NA	H_2_O, Mn^2+^, Ni^2+^, Cu^2+^, Na^+^, K^+^, Mg^2+^, Zn^2+^, Ca^2+^, Co^2+^	NA
[120]	Photoluminescence	Carbon dots	0–300 μM	0.12 μM	Ba^2+^, Ca^2+^, Cd^2+^, K^+^, Mg^2+^, Na^+^, Li^+^, Zn^2+^, Ni^2+^, Al^3+^, Mn^2+^, Fe^2+^, Hg^2+^, Sr^2+^	NA
[116]	Photoluminescence	Amino-functionalized graphene quantum dots	0–100 nM	6.9 nM	Al^3+^, Ag^+^, Co^2+^, Cd^2+^, Ni^2+^, Mg^2+^, Mn^2+^, Pb^2+^, Zn^2+^, Fe^2+^, Fe^3+^, Hg^2+^	Human lung cells
[121]	Photoluminescence	Nitrogen-doped carbon dots	10 μM–0.4 mM	10 μM	Fe^3+^, Fe^2+^, Zn^2+^, Hg^2+^, K^+^, Na^+^, Ag^+^, Mn^2+^, NH_4_^+^, Pb^2+^, Cd^2+^, Ni^2+^, Au^3+^, Mg^2+^, Ca^2+^, Co^2+^	Pond water
[122]	Photoluminescence	Polydopamine	1–1000 nM	1 nM	Na^+^, K^+^, Mg^2+^, Fe^3+^	NA
[123]	Photoluminescence	Polyethylenimine-capped carbon quantum dots	0.3–66.6 μM	115 nM	Co^2+^, Ca^2+^, Ni^2+^, Mn^2+^, Hg^2+^, Pb^2+^, Ba^2+^, Cd^2+^, Fe^3+^	River water
[124]	Photoluminescence	Graphene quantum dots	0–0.2 mM	0.33 µM	Cr^3+^, Ba^2+^, Ca^2+^, Cd^2+^, Co^2+^, K^+^, Mn^2+^, Ni^2+^, Pb^2+^, Zn^2+^, Fe^3+^, Ag^+^, Hg^2+^	Tap water
[147]	Surface plasmon resonance	Molecularly imprinted nanofilm	0.04–5 μM	0.027 µM	Fe^2+^, Cd^2+^, Li^+^, Ni^2+^, Pb^2+^	Artificial plasma and urine
[148]	Surface plasmon resonance	Molecularly imprinted nanoparticles	0.1–100 nM	NA	Ni^2+^, Zn^2+^	Artificial urine and serum
[149]	Surface plasmon resonance	Peptide-modified film	800 pM–100 μM	0.1 ppb	NA	Tap water
[150]	Surface plasmon resonance	Nanocrystalline cellulose-modified composite film	0.01–60 ppm	0.01 ppm	NA	NA
[151]	Surface plasmon resonance	Peptide-immobilized	1 × 10^−12^–1 × 10^−6^ M	0.44 pM	Mg^2+^, Ca^2+^, Zn^2+^, Pb^2+^, Mn^2+^, Ba^2+^, Ni^2+^, Co^2+^	NA
[152]	Surface plasmon resonance	Indium tin oxide film-coated gold nanoparticles	10^−11^–10^−5^ M	5 × 10^−12^ M	K^+^, Fe^2+^, Pb^2+^, Co^2+^, Zn^2+^, Ni^2+^, Cd^2+^, Ag^+^, Hg^2+^	Tap and river water, milk
